# Back to the Suture: The Distribution of Intraspecific Genetic Diversity in and Around Anatolia

**DOI:** 10.3390/ijms12064080

**Published:** 2011-06-20

**Authors:** Rasit Bilgin

**Affiliations:** Institute of Environmental Sciences, Boğaziçi University, Bebek 34342, Istanbul, Turkey; E-Mail: rasit.bilgin@boun.edu.tr; Tel.: +90-537-988-4734; Fax: +90-212-257-5033

**Keywords:** biogeography, glacial refugia, ice age, phylogeography, pleistocene, suture zone

## Abstract

The effect of ice ages in speciation and diversification is well established in the literature. In Europe, the Iberian, the Italian and the Balkan peninsulas comprise the main glacial refugia, where the subsequent re-population of Europe started. Though not studied as extensively, Anatolia has also been hinted to be a potential glacial refugium for Europe, and with its proximity to the Caucasus and the Middle East at the same time, has potential to exhibit high levels of intraspecific diversity. The more ubiquitous use and cheaper availability of molecular methods globally now makes it possible to better understand molecular ecology and evolution of the fauna and flora in the genetically understudied regions of the world, such as Anatolia. In this review, the molecular genetic studies undertaken in Anatolia in the last decade, for 29 species of plants and animals, are examined to determine general phylogeographic patterns. In this regard, two major patterns are observed and defined, showing genetic breaks within Anatolia and between Anatolia and the Balkans. A third pattern is also outlined, which suggests Anatolia may be a center of diversity for the surrounding regions. The patterns observed are discussed in terms of their relevance to the location of suture zones, postglacial expansion scenarios, the effect of geographic barriers to gene flow and divergence time estimates, in order to better understand the effect of the geological history of Anatolia on the evolutionary history of the inhabitant species. In view of the current state of knowledge delineated in the review, future research directions are suggested.

## 1. Introduction

The effects of ice age glacial maxima in causing intraspecific genetic differentiation of terrestrial biota have been well established in the literature. Observations of this phenomenon have been documented on almost all continents. In South America, the distribution of intraspecific diversity has been attributed to differentiation in refugial areas during ice ages, with the isolation and subsequent differentiation processes being referred to as “speciation pumps” [1]. In Africa, consecutive phases of glacial contractions and expansions are considered to have existed during the Pliocene and Pleistocene, with the exceptionally dry-cold spells creating refuges in montane areas in the form of small isolated forests [2]. Late Quaternary changes have also caused substantial geographic range shifts and phylogeographic breaks for various animal and plant species in North America [3].

In terms of the intensity of such research, Europe is probably the continent where the phylogeography of various taxa have been investigated most thoroughly [4,5] in the context of isolation during the ice ages. Studies on various animal and plant species in Europe indicate that, during the Pleistocene and Pliocene, the three main peninsulas (the Iberian, the Italian and the Balkan) acted as refugia from glacial conditions, sheltering various species during the glacial maxima, but at the same time they isolated populations of species, culminating in their genetic differentiation [6].

In Europe, subsequent post-glacial range expansions from these peninsular refugia affected the distribution of intraspecific genetic diversity for many organisms, resulting in the formation of hybrid zones where two divergent genomes met, and suture zones that formed as geographic clusters of hybrid zones for various species. Remington [7] defines a suture zone as “a band of geographic overlap between major biotic assemblages, including some pairs of species or semispecies which hybridize in the zone”. The definition of suture zones has also been expanded in some recent analyses to also include intraspecific phylogeographic breaks and contact zones. For instance, Swenson and Howard [8], through rigorous tests have statistically shown the convergence of these phylogeographic breaks, hybrid zones and contacts zone in particular regions of North America, forming suture zones.

Focusing on Europe, though not without exceptions, three main paradigms have been outlined corresponding to the general course of expansions of differentiated populations or subspecies, which produced the distribution of hybrid zones, and suture zones where they converged [5]. The grasshopper paradigm represents an initial rapid migration front out of the Balkans and Anatolia, with the colonizing populations limiting the expansion of the Iberian and Italian populations past the Pyrenees and the Alps, respectively, and causing hybrid zones in between. The bear paradigm represents a faster emigration rate out of the Balkans, Eastern Europe and the Iberian Peninsula towards central Europe, with the lineages from the Italian Peninsula not being able to expand and subsequently increase their frequency in the rest of Europe. The third—the hedgehog paradigm—is less common, and represents northward expansion of the three lineages that diverged in the Iberian, Italian and the Balkan Peninsulas, at a similar rate of expansion, without blocking each other, and forming Iberian/Italian and Italian/Balkan hybrid zones.

**Figure 1:**
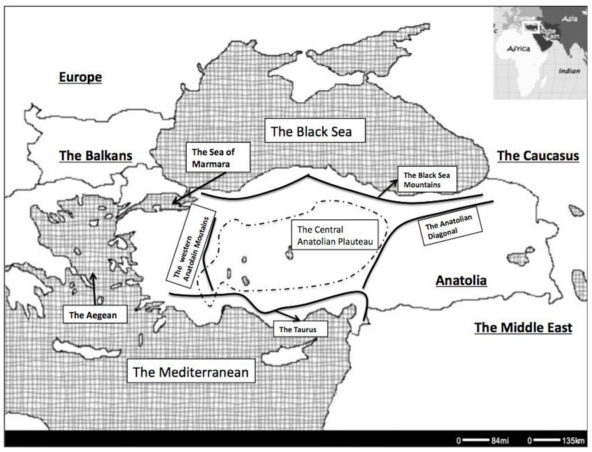
The geographic positioning of Anatolia and surrounding regions, and the major topographic features. The Central Anatolian Plateau, delimited by the major geographic barriers in Anatolia: the Taurus, the Anatolian Diagonal, the Black Sea Mountains, the western Anatolia Mountains. The dashed line represents the area covered by the Central Anatolian Lake System during the Pleistocene [9].

Although the European hybrid and suture zones have been studied in detail, the nature, extent and geographic positioning of the possible zones to the east, firstly in Anatolia and the surrounding regions (Figure 1), have not been investigated as thoroughly. Many studies acknowledge the potential importance of Anatolia as a source and refugium of genetic diversity for European biota, but a detailed analysis of the patterns in the region is lacking. However the recent data generated by the more common use and availability of molecular methods now makes it possible to better explore the molecular ecology and evolution of the fauna and flora in many understudied regions of the world, and Anatolia is no exception.

Politically speaking, Anatolia is a peninsula, surrounded by the Black Sea, the Aegean and the Mediterranean, comprising Asia Minor and the adjacent eastern areas that are within the territory of Turkey. In a biogeographical perspective, it is considered to include Levant, western Iran and northern Iraq [9]. It also contains multiple current and past barriers to gene flow, such as mountain chains (the Anatolian Diagonal (Davis [10]), the Taurus and the Black Sea Mountains), the Central Anatolian Plateau, the Sea of Marmara, and the Central Anatolian Lake system (Figure 1). The formation of the mountain chains in Anatolia can be traced back to the Tertiary, when the northward movement of Europe resulted in the formation of the Alps. This was also when the Central Anatolian lake system, between the Taurus and the Black Sea Mountains, was also initially formed and persisted cyclically until the end of the Pliocene [11]. These various barriers make Anatolia a good candidate for observation of intraspecific genetic differentiation. Being situated between Europe, Asia and Africa, Anatolia also comprises a convergence area for distribution of many species. Placed in such an area of overlap, it hosts parts of three hotspots, the Mediterranean, the Irano-Anatolian and the Caucasus [12], and as such the presence of multiple hybrid zones and local refugial areas within Anatolia can be expected.

In many studies exploring the genetic differentiation in Europe, sampling has been undertaken from the Balkans, but not in Anatolia [5]. Those studies in which samples from Anatolia were included have generally relied on a small number of sampling sites in this region, and followed a broad continental and/or global sampling strategy, to answer phylogeographic questions with samples from a wide geographical range [13,14]. This approach generally involved samples from a few localities *per* region, and Anatolia was no exception. These studies have provided invaluable information, especially in terms of comparing the genetic make-up of populations from Anatolia to those from the Balkans and the Caucasus, and even beyond, but due to the limited sampling could not capture clear patterns of genetic diversity within Anatolia. Although the results hint at the presence of hybrid zones associated with Anatolia, precise mapping of the borders of these zones has not been possible. Rokas *et al.* [15] point out that the relative lack of zones from Anatolia may represent the relatively greater importance of post-glacial latitudinal expansions when compared to longitudinal movements when these hybrid zones are formed in Europe. As Anatolia comprises a southeastern refugium for Europe, the post-glacial dispersals might have not resulted in the formation of many latitudinal hybrid zones in this region. This deficiency is probably also related to the smaller amount of research effort put into studying the phylogeography of species in Anatolia. Whatever its cause, the deficiency has resulted in little estimation of the effect of easterly refugia in shaping the distribution of genetic variation in Europe [15].

In the last 15 years, more elaborate studies with greater sampling intensity were undertaken within Anatolia. Instead of broad geographic sampling strategies, resulting in a small number of sampling points in Anatolia, these recent studies employed a denser sampling regime over a narrower geographic range within Anatolia and the neighboring regions. Consequently, the distribution of genetic diversity within Anatolia and the differences between Anatolia and the neighboring areas have been revealed in greater detail. A variety of organisms, such as amphibians [16], fish [17], and mammals [18] have been evaluated in this manner. The research on these diverse groups of organisms has predominantly indicated the presence of genetic differentiation within Anatolia resulting from isolation in the neighboring Pleistocene refugial areas, such as the Balkans and the Caucasus, with subsequent range expansions into Anatolia after the end of the Pleistocene.

This review outlines the general patterns that emerge when the two categories of research, large and small geographic scales, with their respective sampling intensities, are examined. With this approach, the general patterns of phylogeographic breaks, hybrid and suture zones, postglacial expansion scenarios, the effect of geographic barriers in the region in influencing genetic differentiation, and times of divergence for taxa exhibiting different phylogeographic patterns are investigated. It should be noted that from this point forward, “hybrid zones” are used in a broad sense to refer to areas where two divergent genomes meet, and include phylogeographic breaks/overlaps and contact zones. The species/studies that were reviewed were found based on a literature search that included the Google Scholar and Web of Science databases. As the initial search with Anatolia and genetics gave around 12,000 hits, the search was narrowed down by excluding the following terms: domestication-cultivated-human-language-disease-economic. The resulting, approximately 600, hits were evaluated to be used in the review. Note that only species with natural dispersal modes have been included in this evaluation. Species like the house mouse [19,20], wheat [21], water buffalo [22], honey bee [23] or ocellated skink [24], which have genetic data available from Anatolia, but human-mediated dispersal modes, have been excluded from the procedure of pattern determination undertaken in this review, as they might bias the conclusions to be made regarding natural phylogeographic processes. Also species for which genetic data is available as interspecific phylogenetic comparisons (e.g., water frogs [25], tree frogs [26]) were excluded, as these are not the focus of this study. In a similar manner, studies of species for which genetic data was available from less than five sites from Anatolia (e.g., legless skink [27], Black Sea roach [28], European snow vole [29], and long-eared bat [30]) or those sampled from a very narrow and local geographic range (e.g., Strauch’s spotted newt [31]) have also been excluded.

## 2. Phylogeographic Patterns

**Figure 2:**
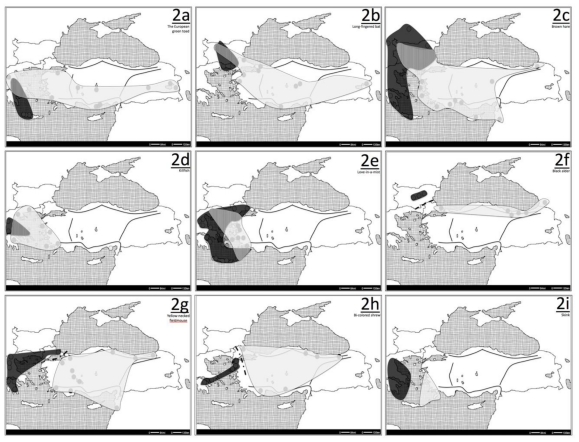
The clade distribution maps for species exhibiting the Pattern I. The white and black shades represent the eastern and western clades, respectively. When present, the grey shaded areas represent the zones of parapatry for the two clades, and the dashed lines represent the allopatric borders between clades. (**a**) The European green toad; (**b**) Long-fingered bat; (**c**) Brown hare; (**d**) Killifish; (**e**) Love-in-a-mist; (**f**) Black alder; (**g**) Yellow-necked fieldmouse; (**h**) Bi-colored shrew; (**i**) Snake-eyed skink.

**Figure 3:**
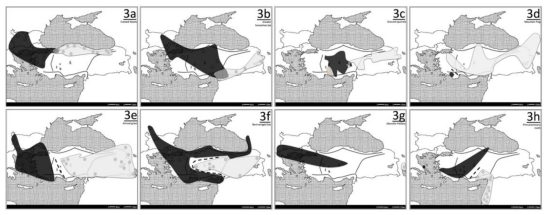
The clade distribution maps for species exhibiting the Pattern II. The white and black shades represent the eastern and western clades, respectively. When present, the grey shaded areas represent the zones of parapatry for the two clades, and the dashed lines represent the allopatric borders between clades. (**a**) Crested newt; (**b**) Greater horse-shoe bat; (**c**) Ground squirrels; (**d**) Mountain frog; (**e**) Annual grass; (**f**) Bent-winged bat; (**g**) Glanville fritillary; (**h**) Pine processary moth.

**Figure 4:**
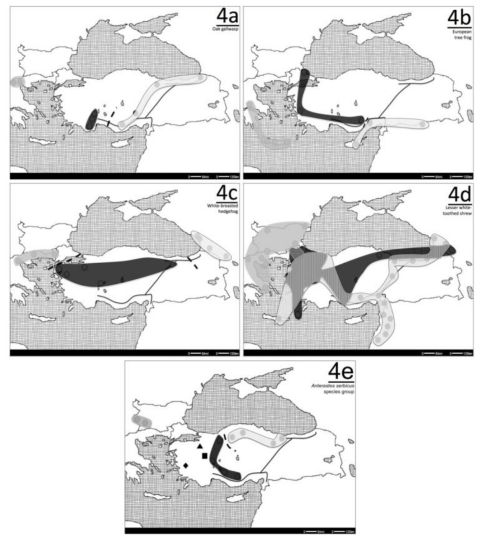
The clade distribution maps for the “other cases”. The white, gray and black shades represent the eastern, central and western clades, respectively. When present, the shaded areas represent the zones of overlap for the different clades, and the dashed lines represent the allopatric borders between clades. (**a**) Oak-gallwasp; (**b**) European tree frog; (**c**) White-breasted hedgehog; (**d**) Lesser white-toothed shrew. (**e**) *Anterastes serbicus* species group—the black triangle, square and rhombus represent locally differentiated populations.

In order to elucidate the general phylogeographic patterns in Anatolia, the intraspecific genetic patterns were evaluated for 29 species for which such data were available (Table 1), based on the criteria outlined above. The ranges for various intraspecific groups (clades) were mapped by extracting the approximate location of sampling sites from the various studies and making subsequent minimum spanning curves around the sites (Figures 2–4). A consistent pattern regarding differentiation of various species involved intraspecific phylogeographic breaks that were longitudinally oriented, differentiating the populations on the east and west. The nature of the boundaries formed by these intraspecific clades/groups defined by these breaks was idiosyncratic for each species. For instance, for the greater horse-shoe bat, *Rhinolophus ferrumequinum*, the minimum spanning curves of the eastern and western clades overlapped, forming a zone of parapatry, a potential hybrid zone, shown as a dashed area in Figure 3b. On the other hand, the eastern and western clades of the bent-winged bat, *Miniopterus schreibersii*, were allopatric, and the curves for each clade do not meet, as the individuals belonging to different clades did not co-occur in a particular site. The boundary between these two clades has been shown with a dashed line (Figure 3f). A general examination of the geographic distribution of the intraspecific genetic diversity in Anatolia as such shows three major patterns. One pattern (pattern I) involves breaks that differentiate the populations in the Balkans and Anatolia. Another set of phylogeographic breaks is seen within Anatolia, differentiating the populations in western Anatolia and the Balkans from those in eastern Anatolia (pattern II). An examination of the intraspecific diversity of the region in a broader geographic context, also including populations from Europe, Africa and Asia also defined another pattern, which I refer to as the “star” (*) pattern. For seven species, the Anatolian region comprises an area of elevated genetic diversity when compared to the surrounding regions, indicating that the populations in Anatolia were the most likely source from which Europe, Africa and/or Asia were subsequently populated, or populations that got differentiated during glacial maxima in these continents subsequently converged in Anatolia post-glacially. This star pattern is not mutually exclusive from the pattern I and pattern II, as the species that provide evidence for Anatolia being a center of diversity for the neighboring continents, usually also show hybrid zones in the areas that define these first two patterns. In addition, some other patterns were observed, though in relatively fewer species, so that they are considered as “special cases”. For instance, in four species the amalgamation of patterns I and II was observed. For these species, phylogeographic breaks were seen both in the Balkans and in central Anatolia such that the Balkan, western Anatolian and eastern Anatolian populations of these species are all differentiated from each other. In one species, intraspecific clades were seen, but they showed an almost complete overlap of geographic distribution within Anatolia. These patterns and cases are discussed in greater detail below.

## 3. Patterns and Representative Case Studies

### 3.1. Pattern I: Anatolian-Balkan Suture Zone

Of the 29 studies included in this review, ten species (including four mammal, two plant, one fish, one insect, one reptile and one amphibian species) showed the presence of a genetic break at the margin between Anatolia and the Balkans, and also within the Balkans (Figures 2a–i, Table 1). This group includes terrestrial, aquatic and volant species. The break can be biogeographically associated with the Aegean trench, which comprises a geographic barrier for various taxa on the Greek mainland and in Anatolia [67], and corresponds to the so-called Rechinger’s line.

In five species the presence of pattern I is relatively clear, with good sampling distribution from the entire Anatolia and the Balkans. Three of these species show a parapatry of the distribution of the eastern and western clades. These are the European green toad (Figure 2a) [36], the long-fingered bat (Figure 2b) [35] and the brown hare (Figure 2c) [38,39]. The two other species with a thorough sampling distribution show an allopatric distribution for their respective eastern and western clades. In the yellow-necked fieldmouse (Figure 2g) [42], the line of allopatry runs parallel to and overlaps with the Bosphorus Strait. On the other hand, in the bi-colored shrew, the line of allopatry is perpendicular to the Bosphorus Strait (Figure 2h) [43] and some of the western haplotypes are seen in western Anatolia and some of the eastern haplotypes in the Balkans. Another species, the European grasshopper also shows a differentiation between the Balkan and Anatolian populations [44], with no obvious differentiation within Anatolia [68]. However, the geographic distribution of these haplotypes have not been explicitly mapped in the original publications [44,68], hence it was not possible to recreate a distributional map for this species in this review.

In one of the remaining four species that fit pattern I, sampling was only done in the Balkans and northern Anatolia. This species is the black alder (Figure 2f) [40], and the western and eastern clades are allopatric with the dividing line being within the Balkans. In remaining three, sampling in Anatolia was only done from Balkans and western Anatolia. In two of these species (killifish [33] and love-in-a-mist [34], Figures 2d and 2e, respectively), there exist parapatric overlap of the eastern and western groups. In the killifish the zone of parapatry is in the Balkans, whereas in love-in-a-mist, it can be seen both in the Balkans and partially in western Anatolia. In the third species, the snake-eyed skink (Figure 2i) [37] there are different clades in western Anatolia and the Balkans, without overlap, however as the sampling distribution is disjunct, a parapatric or allopatric boundary could not be determined. More detailed discussions for two representative case studies are presented below.

### 3.2. Long-Fingered Bat (*Myotis capacinnii*)

Bilgin *et al.* [35] investigated the genetic differentiation of the long-fingered bat, with sampling from 14 locations in Bulgaria, Greece and Turkey, and examining the cyt-*b* region of mtDNA and eight microsatellite loci. The results showed potential isolation of the populations with a mitochondrial break, differentiating the western and eastern populations as two clades, located between Turkey and Greece & Bulgaria (Figure 2b). The divergence was dated to around 500 Kya, in the Pleistocene. The individuals belonging to the two clades were found in sympatry in certain caves in Bulgaria and Greece, indicating a hybrid/contact zone. However, the differentiation was not reflected in the nuclear microsatellites, suggesting that the differentiation in glacial refugia in mtDNA probably did not result in biological speciation. A similar pattern of differentiation of the Balkan and Turkish populations, in mtDNA, was also partly seen in the two large *Myotis* species, *Myotis* and *M. blyhtii*.

### 3.3. Killifish *(Aphanius fasciatus)*

This species is an example of pattern I being observed beyond the terrestrial environment in the brackish waters (Figure 2d). Using RFLP and direct sequencing methods, Triantafyllidis *et al.* [33] evaluated the 16S rRNA, tRNA-Leu, NADH-1 and tRNA-Ile regions of mitochondrial DNA for a total of 158 samples of killifish from 13 sites in Greece and Turkey. The results indicated a high degree of population genetic structure and two main phylogenetic lineages, divided along a western and eastern axis, with a hybrid zone passing through Greece. The eastern group was comprised of the populations from water bodies draining into the Aegean, and the western group was comprised of localities draining into other areas in the Mediterranean. Interestingly, based on the study of Hrbek and Meyer [32], also using mitochondrial DNA, a sample from southern Turkey was seen to be more similar to the western group, indicating a potentially more complex evolutionary history within Anatolia. The mean sequence divergence between the two lineages was 3.45%, which corresponded to a divergence date of about 4 Mya. The results of these two studies suggest that vicariant events affected the evolution of the whole *Aphanius* genus [32,33].

## 4. Pattern II: Intra-Anatolian Suture Zone

The presence of a suture zone within Anatolia can be associated with refugial areas in western and eastern Anatolia [9]. Also postglacial expansion from refugia located more to the west, out of the Balkans, and more to the east from the Caucasus and the Caspian Sea [69], or even beyond are possible. Out of the 29 species investigated, eight showed the presence of potential hybrid zones within Anatolia (Figures 3a–h, Table 1). The species included three mammals, two insects, two amphibians and one plant.

In three species, the divergent clades in the east and west showed a parapatric distributional overlap within Anatolia. These were the crested newt (Figure 3a) [52], the greater horseshoe bat (Figure 3b) [46,47] and the ground squirrel (Figure 3c) [45]. In four other species, although distributions of the divergent clades came very close to each other, there was no evidence for an overlap. In the annual grass *Hordeum* spp. [54], the bent-winged bat [48,49,50,51] and the pine processary moth [55] (Figures 3e, 3f and 3h, respectively), the border of the allopatric distributions passes through central Anatolia. In the mountain frog (Figure 3d) [16], this boundary is within southwestern Anatolia. Finally, in Figure 3g, the pattern for the Glanville fritillary moth [55] can be seen. In this species, as the western and eastern clades were geographically far from each other (in central Anatolia and Iran, due to a sampling bias), a zone of allopatry or parapatry was not indicated. The details for two of the representative species are presented below.

### 4.1. Ground Squirrels *(Spermophilus* spp.*)*

As an example of a species complex with parapatric overlap within Anatolia, ground squirrels, *Spermophilus* spp., can be given. In this complex, Gündüz *et al.* [45] defined a new species (*S. taurensis*) in the Taurus Mountains within Anatolia. They took samples from 183 individuals and 86 localities, and used complete tRNA-Thr, tRNA-Pro and cytochrome-b, and partial D-loop, and X and Y chromosome sequences. The complete molecular data set, combined with morphology supported the diagnosis of the Taurus populations as a separate biological species. The cytochrome-b data provided evidence for the presence of five phylogroups within Anatolia. The authors suggest that these lineages correspond to differentiation in local refugia within Anatolia, and lack of any significant dispersal subsequently. A closer examination of the phylogenetic trees indicates two main clades with lineages 1, 2 and 3 being reciprocally monophyletic with lineages 4 and 5. Mapping the geographic distribution of these clades shows a main parapatric zone within central Anatolia, suggesting a western and eastern refugial area for these two clades, presumably on the two sides of the Anatolian Diagonal (Figure 3c).

### 4.2. Bent-winged Bat *(Miniopterus schreibersii)*

The bent-winged bat, *Miniopterus schreibersii*, is one of the species that shows an allopatric distribution of intraspecific clades. This species was investigated with samples from Bulgaria, Greece, Turkey, Georgia, Armenia and Iran and utilizing mtDNA, microsatellite and morphological data [48–51]. The mtDNA results indicated a western and eastern division, but with a slight twist. The “western” clade was predominantly found to the east in humid coastal regions, whereas the “eastern clade” was predominantly found entirely in drier inland areas (Figure 3f). This suggests that climate might have played a role in the differentiation of these clades, or that the glacial climatic preferences of the ancestral populations were conserved in the more recent generations. The divergence was dated to around 230 Kya, in the Pleistocene. The results also indicated significant genetic differentiation in microsatellites. Statistically significant differences were also seen in the morphology of the clades, measured as the forearm length. The bats in the eastern clade had longer forearms than those in the western clade [49,51]. If in any cave, individuals belonging to the two different clades were to be found in sympatry, this would indicate that the two clades represent two separate biological species.

## 5. The Star (*) Pattern

Another pattern category can be defined for seven species that were investigated in a broad geographic context, with populations from Europe, Asia and sometimes Africa being included, in addition to those from Anatolia. These studies indicated that Anatolia is probably the center of origin for the species. Sometimes this pattern overlaps with the previous two patterns; as the species that have hybrid zones in the region also exhibit high levels of diversity. These have been represented with a star next to the pattern with the corresponding potential hybrid zone in Table 1. For example, for the brown hare ‘I*’ indicates that the species shows a hybrid zone between Anatolia and the Balkans, and also that Anatolia represents the center of origin for this species [38,39]. On the other hand, there are species, such as Alpine rockcress, which do not show the presence of a hybrid zone, but shows the highest levels of diversity in Anatolia [13], and is depicted by a star ‘*’ only. In this regard the brown hare showed the I*, the annual grass (*Hordeum gussoneanum*) showed the II* [54], the oak-gallwasp [15], the brown trout [17,64] and the chub [65] showed the (I&II)* patterns (cases of species exhibiting both patterns I &II, but also Anatolia being a center of diversity). In addition, the European ash (*Fraxinus angustifolia*) [66] fits the pure star pattern, without any breaks within Anatolia or between Anatolia and the Balkans. The details for two species are presented below for the star pattern.

### 5.1. Oak-gallwasp *(Andricus quercustozae)*

In their study on the oak-gallwasp, Rokas *et al.* [15] sampled 42 locations from Morocco, continental Europe and Anatolia. They sequenced 47 individuals at the mitochondrial cytochrome b gene, and also screened 609 individuals at 12 allozyme loci. Both analyses showed a deep split (7 Mya) between the northeastern/central and the southwestern Anatolian populations (Figure 3a), although the clustering pattern of the central Anatolian populations differed in the two methods. In the sequence analysis, the central Anatolian haplotypes clustered with those from Europe, whereas in the allozyme analyses they clustered together with the populations from northeastern Turkey. In general the allozyme data seemed to provide higher resolution than the sequence data, with refugial populations in Iberia, Italy and Balkans. The allozyme allele frequency data also indicated gene flow from Anatolia to the populations in Greece and Italy. The results showed that the highest levels of genetic diversity among the populations sampled were found in Anatolia, suggesting that this region comprised a center of origin for the European populations, and the source for the pre-Pleistocene colonization of Europe.

### 5.2. Alpine Rockcress *(Arabis alpina)*

Koch *et al.* [13] investigated the phylogeography of the Alpine rockcress, *Arabis alpina*, using sequence data from *trn*L-F chloroplast DNA and the ITS of nuclear encoded ribosomal DNA regions. Samples were collected from 142 locations, including all European mountain systems, the Canary Islands, North Africa, eastern Africa, the Arabian Peninsula, the Middle East and Anatolia. The data suggested that the region between the Balkans, the Caucasus and the Middle East (in essence Anatolia) represents the center of origin for *Arabis alpina.* The date of origin of the species in Anatolia was calculated to be around 2 Mya, with three migration fronts expanding out. These included one group migrating to east African mountains, through the Arabian Peninsula. A second group gave rise to the European and northwest African populations. The third group was centered in Asia, and came in secondary contact with the east African populations. Although the species is also found in northern and eastern Anatolia, these regions were not sampled in this study of Koch *et al*. Therefore, further sampling in these regions will be necessary to determine the presence and absence of a hybrid zone in Anatolia.

## 6. Special Cases

Other than the species that fall under the above patterns I and II, in five species, a juxtaposition of Pattern I and Pattern II can be seen. In the oak gallwasp (Figure 4a) [15] and the European tree frog (Figure 4b) [59], the Balkan and western Anatolian populations are differentiated from each other, however a zone of parapatry or allopatry could not be determined, due to sampling gaps. However, between the western and eastern Anatolian samples, a zone of allopatry within central Anatolia can be seen for these two species. On the other hand, in the white-breasted hedgehog (Figure 4c) [60–62], a break is seen between the Balkan and Anatolian populations that coincide with the Bosphorus, suggesting that the strait may be a barrier to gene flow for this species. There is another line of allopatry in this species that is in eastern Anatolia, separating the Anatolian and the Caucasus populations. In addition to these cases, the lesser white-toothed shrew shows a modified version of pattern I (Figure 4d) [56–58]. In this species, intraspecific breaks where two of the three groups have complete overlap within Anatolia, and one of these groups has a parapatric distributional overlap with a third clade in the Balkans. Finally phylogroups within *Anterastes serbicus* species group [63] (Figure 4e) show local differentiation within different regions of Anatolia and between Anatolia and Bulgaria, as another example to a special case.

## 7. Discussion

### 7.1. Suture Zones

**Figure 5:**
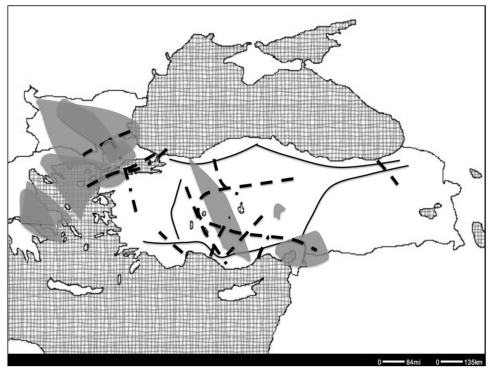
The suture zones as defined by the overlap of the paraptric zones (gray shades) and allopatric borders (dashed lines) of the clades in Figures 2, 3 and 4.

As mentioned above, in a relaxed sense, suture zones can be defined as areas where multiple hybrid zones, contact zones or phylogeographic breaks meet. The mapping of various allopatric and parapatric phylogeographic breaks and from different species (Figures 2, 3 and 4) onto the same map (Figure 5) outlines the presence of two suture zones, one between the Balkans and Anatolia and another within Anatolia. These suture zones show that although the patterns of differentiation and geographic orientation of intraspecific groups are idiosyncratic for each taxon, general patterns do emerge due to the glacial history of the region.

The prevalence of two suture zones associated with Anatolia suggests that the conditions for maintaining these zones are being satisfied for multiple taxa. The point that these intraspecific lineages do not geographically mix is interesting considering the wide time ranges of the split of these clades. Clades that split during Pleistocene show very similar distribution patterns with clades that diverged during the Pliocene. This discrepancy suggests that the interglacial periods in the Pliocene and Pleistocene did not cause the mixing of the divergent clades. There are two possible scenarios regarding the maintenance of the distribution we observe. One is that a narrow hybrid tension zone between the two clades was preserved through the classical cases of hybrid unfitness [5]. However, the initial phase of isolation also had to be long enough for enough differentiation to accumulate to cause hybrid unfitness. It is also possible that after clades started to diverge, the previous interglacials did not provide enough time for the ranges of the divergent populations to expand, even though there might not have been any hybrid unfitness. Hence although the populations were isolated in different refugia at various glacial maxima, the interglacial periods could have caused oscillatory range expansions not culminating in range overlaps of the western and eastern clades for any given species.

Considering that the current distribution of the various intraspecific clades was a result of the expansion after the end of the last glacial maximum, around 15,000 years ago, the diverging clades in each species would have had to not be mixed in the previous interglacials. If any mixing took place in the previous interglacials (for instance, a western clade expanding way to the east and mixing with the eastern clade) then the geographic orientation of the clades would not be as clear cut as it is observed now. As the previous interglacials in the Pleistocene lasted around 20,000–26,000 years [70], which is more than the current 15,000 years since the last glacial maximum and can be considered as a benchmark timeframe under which two divergent clades can meet, the pre-Holocene interglacials would have provided enough time for the ranges of the divergent clades to overlap and mix. However, we do not observe evidence for such a mixture. Hence, rather than non-meeting oscillatory movement of divergent clades, the hybrid tension zones, formed by hybrid unfitness could be the dominant mechanism that did not allow the mixing of the divergent clades across their respective suture zones. Hybrid unfitness has proven to be an important causal factor in the formation of hybrid zones and speciation for many species [71]. With its geographical location between glacial refuge areas, Anatolia can be a region where hybrid unfitness is a potentially dominant mechanism in maintaining intraspecific boundaries, which might ultimately result in or resulted in speciation. More detailed investigations in the zones of overlap between these intraspecifically divergent clades will be necessary to determine the effect of hybrid unfitness in maintaining the disjunct distributions that are observed.

### 7.2. Postglacial Expansion Scenarios

Based on genetic data, the role of the Balkan peninsula as a glacial refugium and source of postglacial colonization of Europe has been well established in the literature [5]. Two Anatolian glacial refugia, one in western Anatolia and one in eastern Anatolia, have also been suggested based on non-genetic data [9]. The lake system present in central Anatolia during the Pliocene (Figure 1), and the inhabitability of the Central Anatolian Plateau in the glacial maxima during the Pleistocene [72] present themselves as potential geological causes for intraspecific differentiation in these epochs. The differences between the Balkan and Anatolian populations can mainly be associated with the formation of the Aegean in the late Pliocene [9]. The presence of intraspecific suture zones between Anatolia and the Balkans (Pattern I) and within Anatolia (Pattern II), supports these geological scenarios. In total, 26 out of the 29 studies reviewed here showed some sort of intraspecific genetic break in either one or both of these suture zones. These patterns were seen differently in various groups of species; for instance, some fish species (e.g., killifish) fit Pattern I, whereas in others (e.g., chub) we see evidence for both pattern I and pattern II. Pattern I was observed for a Vespertilinoid bat (Bent-winged bat) and a Rhinolophid bat (Greater Horse-Shoe bat), but Pattern II was observed for another Vespertilionid bat (Long-fingered bat). Pattern I was observed in a plant, a fish and a mammal. These results suggest that commonalities cannot be seen in terms of life histories of the organisms and patterns cut through phyla.

It is likely that in the species that fit both pattern I and II, a refugial population survived in western Anatolia, as well as the Balkans (and possibly more to the west), and eastern Anatolia (and possibly more to the east). Consequently, the western Anatolia acted as a buffer, limiting the postglacial dispersal of the Balkan and the eastern Anatolian populations. In species that fit Pattern I, of only the Balkan-Anatolian suture zone, either no refugial populations existed in western Anatolia, or the ones that existed went extinct. Subsequently, this area could have been colonized from the eastern Anatolian, Caucasian or Iranian refugial populations. As an alternative explanation, it is also possible that the western Anatolian population expanded postglacially to the east, and the eastern Anatolian population went extinct. In terms of the geographic placement of hybrid zones, when two differentiated clades meet with each other, they form tension zones, which cannot be easily be penetrated by the other once it is formed [73]. Assuming that post-glacially the Balkan, the western Anatolian and eastern Anatolian populations started their expansion at similar geological times, it seems more plausible that the western Anatolian, rather than the eastern Anatolian population expanded to meet with the Balkan populations. If this was not the case, and the western Anatolian population had gone extinct, we would probably see hybrid zones pre-dominantly within Anatolia (pattern II), with the Balkan populations expanding into Anatolia.

For Pattern II, with one suture zone existing in Anatolia, the explanations are slightly more straightforward. With the eastern clades having originated in eastern Anatolia, the Caucasus or south of the Caspian Sea, the western clade either originated in western Anatolia or in the Balkans. Consequently the two clades expanded and met around central Anatolia. As the western Anatolian and the Balkan populations are genetically similar to each other, it is likely that only one panmictic population survived in these regions, for each species. For any given species under this category, it is possible that there was only a Balkan population with western Anatolia initially vacant during the ice age, which was subsequently colonized postglacially; or *vice versa*.

### 7.3. Geographic Barriers

One commonality in the various studies seems to be about the effects of geographic barriers on intraspecific differentiation. In most of the cases, clades of species were seen on both sides of a barrier such as the Taurus, the Anatolian Diagonal and the Sea of Marmara, implying that the geographic barriers probably did not cause differentiation of populations, or limit their postglacial expansions. In only three cases did the distribution of genetic differences overlap with a geographic barrier. These species were the yellow-necked fieldmouse (Figure 2g), the white-breasted hedgehog (Figure 4b), and the *Anterastes serbicus* species group (Figure 4e), which had distinct clades/phylogroups distributed on either side of the Bosphorus Strait, which appears to have acted as a barrier to gene flow. Also, for the greater horse-shoe bat*,* Rossiter *et al.* [47] suggested that the Sea of Marmara might be a barrier to gene flow, indicating a break that fits Pattern I. However, more thorough sampling in Anatolia by Bilgin *et al.* [51] suggested that this break passes from within Anatolia, in concordance with Pattern II, and provided no evidence of the Sea of Marmara or other geographic barriers causing genetic differentiation between populations of this species.

Hence, although ice ages seem to have caused genetic differentiation within species, during their subsequent postglacial range expansions, these species seem to have gradually dispersed over these geographic barriers with time. As speciation or genetic differentiation generally requires allopatric separation [74], the point that various intraspecific clades are not currently divided due to geographic barriers supports the view that intraspecific differentiation in Anatolia is due to isolation in the form of historically impassable/uninhabitable areas in the region (in this case the Central Anatolian Plateau and/or lake system), separating the refugia. It is possible that the corridor referred to as the “Taurus way” [75], connecting the Anatolian Diagonal, the Taurus and the western Aegean mountains may have been instrumental in the dispersal of some of these species, especially those adapted to colder climates. This explanation does not completely support the view proposed by Demirsoy [11] and Çıplak *et al.* [76] that geographic barriers, especially the Anatolian Diagonal, served as a barrier causing differentiation of taxa at the species and subspecies level. The effect of the Anatolian Diagonal may be more important for species with limited dispersal potential (e.g., insects of the family Pamphaginae) as pointed by Demirsoy. However, for the species reviewed here, except the three mentioned above, the multiple chains of mountains including the Anatolian Diagonal, and the Sea of Marmara, do not seem to have been impermeable boundaries for the postglacial expansion of populations or to have significantly contributed to the differentiation of the populations.

### 7.4. Times of Divergence

Before the utilization of genetic markers, reconstruction of the temporal dimension of invasion processes in Anatolia was not possible due to lack of sufficient data, even for mammals with their rich fossil record [77]. The genetic data now available helps us to infer some of these details of the recolonization of Anatolia. Still, our power of inference is limited. For instance, there was no concordance in divergence times of species or subspecies among the different organismal groups, which was also the case in many European species [5]. In Anatolia, of the 19 species for which clade divergence times were computed, 10 were dated to within the Pleistocene, and nine to the Pliocene. Some patterns were seen in certain groups, nonetheless. For instance, clades in five out of seven mammals (except the yellow-necked fieldmouse and the white-breasted hedgehog) were observed to have split during the Pleistocene. Also, regarding the species that show pattern II, of differentiation within Anatolia, the Pliocene split could have been expected to be associated with the freshwater species, as the brackish water Pliocene lake system in central Anatolia [9] could have acted as a barrier to gene flow. The species with divergences dating to the Pliocene included two anuran species (the mountain frog and the European great toad), one urodeles species (the crested newt), as well as two fish species (killifish and chub) that live in freshwater, suggesting that this lake system could have been instrumental in the differentiation of these species.

Comparing the divergence times of species that were studied in Europe with the estimates for the Balkan/Anatolian region shows compatibility in some species, and helps to elucidate the details of the evolutionary history of these species at a larger geographic scale. Two species that were investigated in both Europe and the Balkan/Anatolian transition zone also had their divergence dates estimated. In the hedgehog, *Erinaceus spp.*, the times of divergence calculated around 3–6 Mya for the Iberian, Italian and Balkan peninsulas are comparable to the divergence date (~3 Mya) within Anatolia. This shows that the entire split of the species into various lineages took place in the same epoch, the Pliocene, in a parallel manner. In the lesser white-toothed shrew, *C. suaveolens*, the European split took place about 1.72 Mya (1.40–2.23 Mya). The split between western Anatolia and Europe was dated to around 940 Kya (760 Kya–1.21 Mya). Hence, in this species, the split in Anatolia took place later than that in Europe. It is possible that the expansion of the European population was halted in the Balkans due to the Anatolian population, which had already differentiated in isolation during the Pleistocene and already existed in Anatolia when the European populations arrived.

### 7.5. Anatolia as a Center of Diversity

Non-genetic methods of analyses, such as species richness maps [78] and presence of three hotspots in Anatolia have shown it to be an area with high levels of species diversity. In a parallel manner, Anatolia comprises a region exhibiting high levels of genetic diversity. As Rokas *et al.* [15] indicate, important populations of many western Palaearctic taxa are indeed located to the east of Europe, and “although these more easterly populations are rarely considered, they may not only represent significant centers of genetic diversity, but also potential origin of populations now occupying Europe”. This was seen to be the case for some species that were studied with broad genetic sampling from Europe and other neighboring regions. The brown trout, the oak gallwasp and Alpine rockcress were species that provided evidence for Anatolia being such a center of diversity. It is also likely that the other species that fit to patterns I and II, and which were investigated at local scales, if studied at more continental geographic scales, could provide evidence for high levels of genetic diversity in Anatolia, when compared to the neighboring regions, and give further support to the region being a center/origin of genetic richness. Also, even at a local scale, the suture zones also define areas where divergent clades meet, and by definition comprise regions containing high levels of genetic diversity. This would also have important conservation implications, as these suture zones can be prioritized as areas for protection of the populations of individual species.

### 7.6. Conclusions and Future Research

Although generally considered to be a single refuge, the idea of “refugia within refugia” have been proposed formerly for the Iberian peninsula [79]. This view suggested that multiple smaller areas have existed within Iberia, where isolated populations persisted, resulting in elevated levels of cryptic genetic diversity [80]. The overview of the genetic studies in Anatolia undertaken in this review suggests that, in a similar manner and as suggested by Medial and Diadema [81] and Çıplak *et al.* [76], treating Anatolia as a single refugium is an over-simplification. In addition to some very local differentiation such as that seen in the *Anterates serbicus* group, an examination of intraspecific genetic differentiation from various taxa show at least two main refugial regions in and/or around Anatolia, in the west and east, with the shapes of hybrid zones and their geographic patterns changing based on individual species. The patterns observed for various species also suggest that isolation of populations of species during the ice ages and their subsequent genetic differentiation is the most over-arching process of intraspecific evolution in Anatolia and its surrounding regions. The numerous geographic barriers present in the region have not necessarily acted as major impediments to postglacial expansion for most of the species. There is also evidence for Anatolia being a general hotspot of genetic diversity in a continental perspective.

Further research in Anatolia should also include species like the brown bear and the oak, which have been extensively studied in Europe, to complete the general picture in terms of the intraspecific evolution of these species more comprehensively. On the flip side, it will also be informative to investigate species with European distributions that have been examined regionally in Anatolia (e.g., bent-winged bat, mountain frog), at a more continental scale. These complementary lines of research will help to complete our understanding of the evolutionary differentiation of populations in Asia Minor and Europe during the ice ages, and explain the origins of the high genetic and species diversity found in Anatolia. Thorough geographic sampling in Anatolia will also help to avoid inferential problems, such as those mentioned above for the greater horse-shoe bat, where the denser sampling within Anatolia indicated that the Sea of Marmara was unlikely to be a barrier to gene flow, an earlier suggestion made based on limited distribution data from Anatolia.

Due to its location at the junction of Europe, the Caucasus and the Middle-East, the biogeography of Anatolia is also affected by the Middle Eastern geographic history. For instance, research has shown that the Israeli populations of some species are differentiated from the Balkan/Anatolian ones [62]. Concordantly for the species investigated in this review, it will be interesting to make comparisons with those in the Caucasus and the Middle East, in a broad sense, including populations from Georgia, Iran, Syria, Israel, Jordan. Organismally speaking, it will be informative to look at other species in genera like the bat *Myotis*, where different species have shown congruent patterns (e.g., both *Myotis myotis*/*blythii* and *Myotis capaccinii* conform to pattern I). Also, the possibility of multiple smaller refugia within Anatolia needs to be investigated with more fine-tuned sampling. Working with species that have limited dispersal capabilities, these investigations could pinpoint the exact locations of these local refugia within Anatolia. As Tolkien once said “Little by little one travels far”, and new data being produced in the genetically under-studied regions of the world, such as Anatolia, is advancing the field of molecular ecology by helping come up with general conclusions on the intraspecific evolution of various species and untangle regional details of their biogeographic histories. The missing pieces of a big jigsaw puzzle are being found as we speak and the next little steps are eagerly awaited.

## Figures and Tables

**Table 1:** An overview of the species examined in the review, showing the markers used, age of divergence of intraspecific splits, the pattern and respective references.

Species	Marker	Age of Divergence	Pattern	Reference
Killifish, *Aphanius fasciatus*	RFLPs and mtDNA sequencing	Pliocene (4 Mya)	I	[32,33]
Love-in-a-mist, *Nigella arvensis* alliance	PCR-RFLP	Pleistocene (<1 Mya)	I	[34]
Long fingered bat, *Myotis capaccinnii*	mtDNA sequencing and microsatellites	Pleistocene (500 Kya)	I	[35]
The European green toad, *Bufo viridis* (2n)	mtDNA sequencing	Pliocene (4.8–3.6 Mya)	I	[36]
Snake-eyed skink, *Ablepharus kitaibelii*	mtDNA sequencing	Pliocene (5.9–5.7 Mya)	I	[37]
Brown Hare, *Lepus europaeus*	RFLPs and mtDNA seqeuncing	Pleistocene (490–105 Kya)	I*	[38,39]
Black Alder, *Alnus glutinosa*	RFLPs	N/A	I	[40]
Eurasian shrub, *Frangula alnus*	RFLPs	N/A	I	[41]
Yellow-necked fieldmouse, *Apodemus flavicollis*	mtDNA sequencing	Pliocene (2.4–2.2 Mya)	I	[42]
Bicolored shrew, *Crocidura leucodon*	mtDNA and nuclear sequencing	0.691 Mya (CI: 0.510–0.980)	I	[43]
European grasshopper, *Chorthippus parallelus*	nuclear sequencing	N/A	I	[44]
Ground squirrels, *Spermophilus spp*	mtDNA, sequencing (X and Y chrom.)	N/A	II	[45]
Mountain frog, *Rana macrocnemis*	mtDNA sequencing	Pliocene (2.4 Mya)	II	[16]
Greater horseshoe bat, *Rhinolophus ferrumequinum*	mtDNA sequencing and microsatellites	Pleistocene (350–750 Kya)	II	[46,47]
Bent-winged bat, *Miniopterus schreibersii*	mtDNA sequencing and microsatellites	Pleistocene (170–300 Kya)	II	[48,49,50, 51]
The crested newt, *Triturus karelinii*	mtDNA sequencing	Pliocene (5.5 Mya)	II	[52]
Glanville fritillary, *Melitaea cinxia*	mtDNA sequencing	N/A	II	[53]
Annual grass, *Hordeum gussoneanum*	Chloroplast sequencing, microsatellites	N/A	II*	[54]
Pine processionary moth, *Thaumetopoea wilkinsoni*	mtDNA sequencing, AFLPs, microsats	Pleistocene (1.5–0.5 Mya)	II	[55]
Lesser white-toothed shrew, *Crocidura suaveolens*	mtDNA and nuclear gene sequencing	Pleistocene (940 Kya)	I&II	[56,57,58]
Tree frog, *Hyla arborea*	mtDNA and nDNA sequencing	N/A	I&II	[59]
White-breasted hedgehog, *Erinaceous concolor*	Allozymes and mtDNA sequencing	Pliocene 3 Mya (B-A)	I & II	[60,61,62]
*Anterastes serbicus* group	mtDNA sequencing	Plesitocene, <1.56 Mya	I&II	[63]
Oak-gallwasp, *Andricus quercutozae*	mtDNA sequencing and allozymes	Pliocene (7 Mya)	I&II*	[15]
Brown trout, *Salmo trutta*	RFLPs	Late Pleistocene	I&II*	[17,64]
Chub, *Leuciscus cephalus*	mtDNA sequencing	Pliocene 3–2.5 Mya	I&II*	[65]
Alpine rockcress, *Arabis alpina*	C.plast and nDNA sequencing	N/A	*	[13]
European ash, *Fraxinus angustifolia*	Chloroplast microsatellites	N/A	*	[66]
